# Competing priorities that rival health in adults on probation in Rhode Island: substance use recovery, employment, housing, and food intake

**DOI:** 10.1186/s12889-018-5201-7

**Published:** 2018-02-27

**Authors:** Kimberly R. Dong, Aviva Must, Alice M. Tang, Curt G. Beckwith, Thomas J. Stopka

**Affiliations:** 1 Department of Public Health and Community Medicine, Tufts University School of Medicine, 136 Harrison Avenue, Boston, MA 02111 USA; 2 The Miriam Hospital and Brown University Alpert School of Medicine, Providence, RI USA

**Keywords:** Criminal justice, Probation, Food insecurity, Hunger, Social determinants of health, Homeless, Substance use, Employment

## Abstract

**Background:**

Individuals on probation experience economic disadvantage because their criminal records often prohibit gainful employment, which compromises their ability to access the basic components of wellbeing. Unemployment and underemployment have been studied as distinct phenomenon but no research has examined multiple determinants of health in aggregate or explored how these individuals prioritize each of these factors. This study identified and ranked competing priorities in adults on probation and qualitatively explored how these priorities impact health.

**Methods:**

We conducted in-depth interviews in 2016 with 22 adults on probation in Rhode Island to determine priority rankings of basic needs. We used Maslow’s hierarchy of needs theory and the literature to guide the priorities we pre-selected for probationers to rank. Within a thematic analysis framework, we used a modified ranking approach to identify the priorities chosen by participants and explored themes related to the top four ranked priorities.

**Results:**

We found that probationers ranked substance use recovery, employment, housing, and food intake as the top four priorities. Probationers in recovery reported sobriety as the most important issue, a necessary basis to be able to address other aspects of life. Participants also articulated the interrelatedness of difficulties in securing employment, food, and housing; these represent stressors for themselves and their families, which negatively impact health. Participants ranked healthcare last and many reported underinsurance as an issue to accessing care.

**Conclusions:**

Adults on probation are often faced with limited economic potential and support systems that consistently place them in high-risk environments with increased risk for recidivism. These findings emphasize the need for policies that address the barriers to securing gainful employment and safe housing. Interventions that reflect probationer priorities are necessary to begin to mitigate the health disparities in this population.

## Background

At the end of 2015, one in 37 adults in the United States (or about 2.7% of the population) was under some form of correctional supervision [1], which includes prison, jail, parole, and probation. Adults on probation make up the largest group of individuals under correctional supervision (56%) [1] with 1 in 66 adults in the US under probation supervision by the end of 2015 [2]. Probation is a court-ordered period of community correctional supervision that is generally used as an alternative to incarceration [2].

Adults in prisons and jails have higher rates of chronic disease compared to the general population [3–7] however, little is known about the health of individuals under community supervision, which includes probation and parole. The limited literature on probationers shows that they experience poor health outcomes, such as mental health issues [8], and many engage in HIV-risk behaviors [9–12] and substance use [11, 13–15]. Additionally, in a pilot study of recently released prisoners, Wang et al. found 91% to be food insecure, with 37% reporting not having eaten for an entire day due to lack of money [12]. Contextual and environmental factors, such as policies, programs, or social norms that impact opportunities for employment, housing, and education, are the root causes of poor health outcomes [16], particularly for individuals involved in the criminal justice system. A deeper understanding of how these structural issues influence the health of probationers is needed in order to improve their wellbeing.

Without steady sources of income, the ability to access the basic components of wellbeing--such as housing, food, and healthcare--is compromised, and may contribute to health disparities. Unemployment and underemployment in probationers have been studied as a distinct topic [17–19] but no research has examined all of the components of basic needs in aggregate or explored how individuals under correctional supervision prioritize each of these factors. Because these basic components of wellbeing each influence health and are interconnected, understanding how probationers’ prioritize these needs and their attitudes about them will help to develop effective and relevant interventions to improve their health. Whereas studies of impoverished and vulnerable groups have shown that individuals with material hardships may be forced to make choices about securing basic needs (such as housing and food) over seeking healthcare [20–22], but this has not been explored in probationers. Thus, little is known about competing needs, priorities, and the health status of individuals on probation. The objectives of our study were to identify and rank competing priorities in adults on probation, qualitatively explore how these priorities impact health, and provide perspectives about health concerns and specific attitudes and behaviors that arise in response to competing demands and constrained resources.

## Methods

### Setting and participants

Rhode Island has the second highest rate of community corrections supervision in the nation with 23,595 adults on probation (or about 2.2% of the state population) [2].

We conducted in-depth interviews with English speaking adults (≥18 years) that were under active probation supervision at one office in Rhode Island during June and July 2016. All interviews were conducted in a private room at the probation office without probation officers present. We used convenience sampling to recruit participants in the waiting room of the probation office or by referral from probation officers. We sought to enroll 30% female probationers for this study to mirror the gender distribution in the Rhode Island probation system. A small number (10%) refused to participate at the time of recruitment because the timing was inconvenient with transportation home or other appointments. Fewer than 5 % of the individuals that reported to this office were estimated to be non-English speaking.

### Interviews

The interview guide was designed to explore two content areas: (1) current priorities while on probation (*“Please look at the topics on these index cards [topics read aloud in random order]. Are any of these topics a current priority in your life?”*) and (2) current health concerns (*“What, if any, health concerns do you have?”)*. To assess potential risk for chronic disease, participants were asked to report their height and body weight. Body mass index (BMI) was calculated for participants and standard cutoffs were used to identify individuals as overweight or obese [23]. One researcher (KD) conducted all interviews. All participants consented to audio recordings of the interviews. Each interview lasted an average of 30 min. Interviews were transcribed verbatim by one research assistant and the transcriptions were validated by a different research assistant. Interviews continued until themes reached saturation. The study was reviewed and approved by two Institutional Review Boards, The Miriam Hospital and the Rhode Island Department of Corrections. Each participant provided consent to participate in the study and received a $25 gift card.

### Ranking of priorities

A modified rank order approach was used to rank the priorities chosen by the participants [24]. We pre-selected seven needs for participants to rank. The needs chosen were based upon the basic needs (physiological and safety levels) of Maslow’s hierarchy of needs theory [25], which has been used by other studies to understand competing priorities [21, 22]. We hypothesized needs would include food, housing, employment, healthcare, and providing for others. Based on other studies conducted among populations under criminal justice supervision [11–14], we also included substance use and recovery as potential needs. We added an “other” option for the priority ranking exercise to allow participants to identify other needs of importance that were not pre-selected. These “other” priorities could span to other levels of Maslow’s hierarchy. Each need was listed on separate index cards and we asked participants to select the cards that were relevant to their lives, and then to rank the needs from greatest to least importance. Scores were given based upon the participant’s ranking with the highest priority need given a score of one, the second highest priority a two, and so forth. To elucidate the top four priorities, a mean score was calculated for each need based upon the rankings across participants.

### Analysis

Using both the interviewer guide and the literature [11–14], we developed a coding scheme related to our broad study objectives about competing priorities and health concerns. We analyzed de-identified transcripts using thematic analysis [26], and an inductive approach to derive themes from the data that reflected the semi-structured question format. During this initial coding process, additional themes that emerged from the data were added to the coding themes. After the initial development of the coding scheme was completed, the study team convened to discuss the codes and major themes to build consensus and to begin to interpret and analyze preliminary results; consensus was achieved. Identification of concepts and themes were coded using NVIVO11 software (QSR International Pty Ltd., Melbourne, Australia). Following the initial coding of two transcripts, two members of the research team met to review and discuss coding applications and interpretations of divergent data to resolve the minimal differences that emerged as coding progressed. During this process, and as we coded the final few transcribed interviews, it became evident that we were no longer learning about new domains and themes, and that we had reached thematic saturation. To assess reliability, one research team member randomly selected two interviews and reviewed the coding. We identified and interpreted salient themes in the coded data that were consistent among at least three participants to avoid presentation of “outlier” perspectives. Verbatim quotes with the participant’s race/ethnicity, gender, and age are presented to illustrate key themes.

## Results

**Fig. 1:**
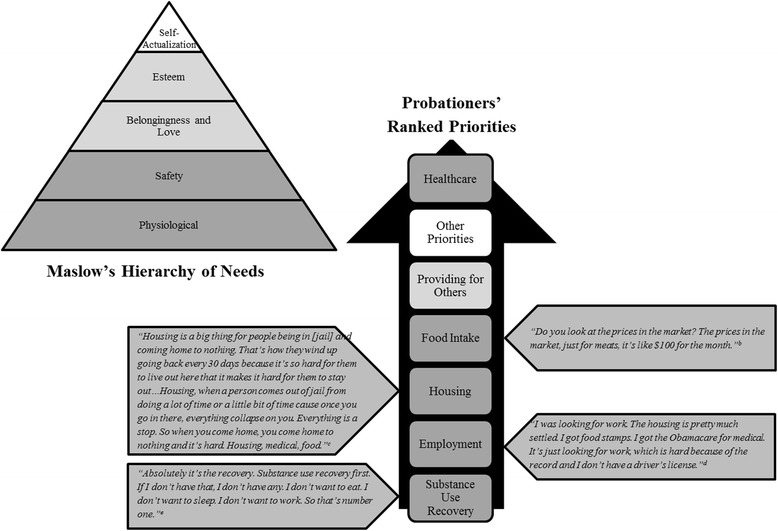
Probationers’ ranked priorities as they relate to Maslow’s hierarchy of needs, Rhode Island, 2016. This figure compares Maslow’s hierarchy of needs (left top triangle) with the ranked priorities of probationers (vertical arrow). Maslow’s basic needs, which comprises the bottom two levels of the pyramid (physiological and safety), were the basis for the pre-selected priorities for our qualitative study. The ranking of the priorities are depicted with the highest ranked priority (substance use recovery) on the bottom of the arrow in order to the lowest ranked priority (healthcare) at the top of the arrow. While most of the ranked priorities correspond with the basic needs of Maslow’s hierarchy, the priority of providing for others corresponds with Maslow’s psychological needs, which consist of “belongingness and love” and “esteem”. The priorities ranked by probationers are shaded in the same color of the corresponding level of Maslow’s hierarchy to demonstrate how these concepts are connected. Because the “other priorities” identified by probationers varied along Maslow’s hierarchy, this priority was left colored white. Qualitative findings present probationers’ perspectives related to each of the top ranked priorities. For the top four priorities, salient quotes support why these priorities were ranked of high importance by probationers are included in the side arrows. ^b^Latina female, 25 years old; ^c^Latina female, 64 years old; ^d^White male, 54 years old; ^e^White female, 33 years old

The demographic characteristics of the 22 adults on probation that participated in this study are provided in Table 1. Participants selected an average of four cards to rank priorities (range 0–7). Mean scores for priority rankings are shown in Table 2. The top four priorities in order from highest to lowest ranking were: substance use recovery, employment, housing, and food intake. “Other” priorities identified from the interviews were transportation/getting a car (*n* = 3), avoid going back to jail (*n* = 2), applying for disability (*n* = 1), and “mental, spiritual, and financial well-being” (*n* = 1). None of the participants ranked “seeking substances” as a priority. Healthcare was ranked the lowest priority by the participants. Figure 1 depicts the order of the priority rankings by participants compared to Maslow’s hierarchy of needs. The themes from the top four priorities and current health status are summarized below. Figure 1 also presents salient quotes that reflect why these top four priorities are ranked high among participants. Additional quotes to support themes are provided in Table 3.

**Table 1:** Selected demographic characteristics of probationers interviewed in Rhode Island, 2016 (*n* = 22)

Characteristics	No (%) or Median (IQR)^a^
Gender Identification	
Male	15 (68)
Female	7 (32)
Age (years)	31 (27, 48)
Race/ethnicity	
Hispanic/Latino(a)	5 (23)
White	17 (77)
Black	5 (23)
Time spent on current probation term (months)	24 (12, 60)
Supplemental Nutrition Assistance Program (food stamps) participation	16 (73)
Anxiety about having enough food^b^	12 (55)
Had dependents (of any age) to provide for	17 (77)
History of illicit drug use (not including marijuana)	10 (45)
Weight status^c^	
Normal Weight	7 (32)
Overweight	6 (27)
Obese	8 (36)

^a^IQR = Interquartile range (25th%ile and 75th%ile)

^b^Anxiety about having enough food was assessed by asking the question, “Tell me about whether or not you go through periods of time when you are not sure you will be able to get enough food”

^c^Determined by Body Mass Index (weight (kilograms)/height (meters)^2^) standard cutoffs which was calculated based upon self-reported height and weight

**Table 2:** Priorities ranked in a study of adults on probation in Rhode Island, 2016 (*n* = 22)

Priority	No (%) That Ranked the Priority	Mean Rank Score (SD)	No (%) Ranking the Priority Highest (Score = 1)	Range of Ranking Scores
Substance Use Recovery	9 (41)	1.78 (1.30)	6 (27)	1–4
Employment	13 (59)	2.15 (1.14)	4 (18)	1–5
Housing	14 (64)	2.36 (1.15)	4 (18)	1–5
Food	15 (68)	2.60 (1.35)	4 (18)	1–5
Providing for Others	12 (55)	3.00 (1.91)	4 (18)	1–6
Other	7 (32)	3.29 (2.36)	2 (9)	1–7
Healthcare	12 (55)	3.75 (1.35)	1 (5)	1–5

SD = standard deviation

**Table 3:** Additional thematic quotes from probationers in Rhode Island, 2016 (*n* = 22)

Priority	Participant	Quote
SUBSTANCE USE RECOVERY		
Why a priority	*White male, 32 years old*	*“Substance use and recovery. Because I got to have my Suboxone to be able to go to work to get the food to have the house.”*
Substance use is not effective for coping	*Black male, 26 years old*	*“Well, I tried it [alcohol] before but it doesn’t work. It makes everything worse. You need money to do these things, you know? I’m not really in that predicament to be doing things like that. I did it before…but it got me on probation.”*
	*White male, 58 years old*	*“Living in pain, not taking pain pills, man, is a challenge. Because I don’t want to get addiction. Because once you take the pills, the pain goes away, you know? Then you got to take more pills for the pain to go away and then you keep taking pills. Next thing you know, you’re going to have a habit, man. And then what happens when I get kicked off my doctor for taking too many pills or something? And then I’m forced out there in the streets, man, where then kicking a habit.”*
EMPLOYMENT		
Why a priority	*Black male, 26 years old*	*“Like anything else, they’ll help you. Like if you’re homeless or something, they’ll help you. Like the food stamp people or whatever, they’ll help you. But you got to help yourself [with finding a job]…I just keep trying.”*
Barriers to employment	*White male, 32 years old*	*“You try to be honest, like you’re looking for a job. You’re trying to be honest with people and tell them. You don’t want them to find out some other way and act like you were trying to hide it [criminal record] or lie about it because you could end up getting a job and they find out and you get fired the next day. And a lot of people, they tell you, ‘Oh yeah.’ But like a few people, when I went and filled out the application, I said, ‘I mean, I do have a record. It’s been a long time. It’s nothing right now, not much of a big deal.’ And I don’t know, a lot of places, they just (pause). You could tell like once you said [you have a criminal record], ‘OK. Thanks for telling me that,’ but then you never hear back.”*
	*Latina female, 64 years old*	*“Jobs, it’s hard to get out there. Especially us prisoners coming from jail out here. Very hard. Like I said the first thing they do is your background and when they do that and they see you are, they don’t want to call you. So you know, it’s really hard. The main topic here is jobs. Education, cause without the education you’re not going to get nowhere. But still even though if you got the education and you come from jail, it’s still hard.”*
	*Black male, 29 years old*	*“I mean, at the end of the day, you know, your business owner has to protect their business and protect their company…I mean not every apple is bad, but not every apple is good either. So I mean, I can’t really say, ‘Oh, they need to change their policy and allow people with records to work there.’ I just feel like somebody shouldn’t be judged off their criminal record. If you’re on probation, you know, and you get a job, that should be something your probation officer would have to approve of you working there. Not for a job to say, ‘He’s got a criminal record. He can’t do this job. I don’t want to hire him.’…I guess it’s public record. I understand that. But companies shouldn’t be able to judge you off of that. To me, that’s a form of discrimination. That’s how I feel. It’s a form of discrimination. ‘Oh, he’s got a record, so he can’t do this job and he can’t be honest.’ Well, how do you ever want me to change? How do you want people who…you know, I’ve never sold drugs a day in my life and I’m proud of that, but how do you want these guys who are out here selling drugs to not sell drugs, when they get arrested and then they can’t get a job? They’re going to revert back to what they know.”*
Types of jobs available	*Black male, 29 years old*	*“Mostly like labor. That’s what I do. I paint, landscape, warehouse jobs, stuff like that. Move-in jobs.”*
	*White male, 22 years old*	*“I’m doing landscaping out of a company in [masked]. It’s hard work, but it’s work and it pays. So, right now, I’m on. There’s like two crews, like one crew cuts and does all that. My crew, we mulch. I’m saying we take out weeds and we just trim stuff down and make sure it looks nice.”*
Alternatives to employment	*Black male, 40 years old*	*“Sometimes I go look for under the table jobs….Like fix houses or whatever’s needed. Just for the extra money for the pockets. That helps for my house and everything.”*
	*Latina female, 64 years old*	*“Sell drugs. In order for me, because I was by myself with my kids, I didn’t have no other choice but sell drugs and that helped me, you know, because you have three kids, four kids, and whatever they give you in food stamps and it runs out. It’s hard to go out there, you don’t get a job, you don’t have a job, you don’t have a man.”*
	*White female, 33 years old*	*“Last year I did snow removal and that was great, but that was not on the book so it’s hard for me to fill that out on a resume.”*
Second chances lead to employment	*White male, 22 years old*	*“I got blessed. As soon as I got out, my friend said, ‘Listen, you know, I got this job. If you want, talk to my bosses.’ And then when I called them, he said, ‘When can you come in’? So I met with him very early, like 6:30 am. And he’s like, ‘Well listen. Fill this application out and I’ll talk to you.’ I filled it out. He said, ‘When can you start?’ I said, ‘I’ll start right now.’ He said, ‘Alright. Go put your boots on and we’re gonna go out.’ And that’s how it happened. A week after I got out of jail.”*
	*White female, 33 years old*	*“Rhode Island is kind of like you gotta know somebody to get work, especially with some kind of past. You know, to get a chance you basically have to know somebody.”*
HOUSING		
Housing milieu	*White male, 22 years old*	*“Well, I’m staying with a friend right now. She’s actually really cool about it. I really had nowhere to go. She said, ‘Well listen. You can stay here until you get on your feet. But you got to look for an apartment. Just help me with the rent.’ So I’m doing that right now.”*
	*Latina female, 25 years old*	*“But going near [masked], south side. All that. Cause that’s where cheap housing is when you’re paying your own rent. It’s not a good area. People die every other day. The things I’ve seen growing up. It’s not fair.”*
Barriers to housing	*White male, 38 years old*	*“Well actually, our plan is having an apartment together. She [his sister] said move out of her house and so we can get a place together. Because of my probation, what I did was a felony, and most housing don’t accept that.”*
	*Latina female, 25 years old*	*“It’s hard. I’m struggling. I’m supposed to moving [from sober house] by the first and I still haven’t found anything yet. And I’m pregnant and these people have little sympathy. They look up that background. ‘Nope!’ And then it’s like, what do I do? I have somebody else rent for me? Cause that’s what’s going on around here. People are renting other people’s things out because people don’t want to rent to certain people.”*
FOOD INTAKE		
High cost of food	*Latino male, 29 years old*	*“It’s too much money in the food. My kid eat so much, you know? And drink milk, the milk expensive money. It’s crazy.”*
Food insecurity	*Latino male, 27 years old*	*Sometimes it’s like that [he experiences periods of hunger]…Like sometimes, today I’m going to take this gift card [stipend] and go buy some stuff cause my food stamps are running out. So I need this gift card to go get some—a couple things for my house. “He also mentioned that each month, towards the last two weeks, he really tries to make food stretch.”*

### Top four priorities

#### Substance use

Participants described the types of substances used currently and in the past, why substance use was not effective for coping, and community-level observations. The substances that participants reported recovering from included alcohol, cocaine, heroin, and oxycodone. Five participants indicated current marijuana use to relax or de-stress. Some individuals with a history of substance use identified recovery as their first priority and indicated that without sobriety, it was not possible to consider other factors in life.

A prominent theme among participants that emerged from the data regarding substance use was that individuals in recovery recognized that substance use was not an effective way to cope and instead, made existing problems worse.

*“Honestly, [alcohol] was just an escape. It didn’t really provide anything for me. It just provides more problems because once you’re not drunk, everything comes back to reality. And you’ve got to deal with the hangover now and the cost of it…I make smart decisions. But I make stupid decisions at the same time when I’m drunk.”—White male, 22 years old*

*“I’ve tried that [using substances] before, in the past, and it did nothing. Set me back even more.”—Black male, 29 years old*

Several participants spoke in depth about observing substance use in the community and some described that they personally knew people who used substances. Another salient theme that emerged from the coded data was that these experiences of seeing the negative impacts of substance use were reported as reasons to avoid using drugs and alcohol.

*“I myself don’t do drugs. I mean, people who do them, that’s on them…I don’t knock anybody…It’s your life. To each your own…But I don’t…I have substance abuse with my uncles and stuff like that so I’ve seen what it does to people…You don’t want to be like that. Nah. You don’t want to be a crackhead.”—Latino male, 27 years old*

*“My girl, she has a drug problem. She does coke and I don’t like it. I don’t want to be around it.”*—*White male, 58 years old*

#### Employment

Some identified employment as a priority because there were fewer resources available to find a job compared to food or housing, especially with a criminal record. Participants described the barriers to employment, compensation mechanisms to address unemployment, the types of jobs that were available for people with a criminal record, and how some individuals were fortunate to have employers give them a second chance at employment.

The most notable theme regarding about employment was that many participants discussed barriers to employment primarily due to their current probation status or history of incarceration and had stories of experiencing stigma with employers. Another relevant employment theme among probationers was how the lack of employment creates worsening financial decline and the detrimental impact on their lives and families.

*“Employment for ex-cons. You know, they don’t really give you a chance…They make it hard on you. You get out. It puts so much rules and guidelines on you, take time from you, they take money from you, the courts you got to pay restitution and all types of fees and all that. But you don’t have no job and then you can’t support yourself. You’re not eating and you’re not feeding. You’re not doing what you need to do for your kids but they’ll take money from you…It’s crazy.”—Black male, 29 years old*

Many participants indicated that the types of jobs they were eligible or hired for were low wage. Most jobs available for probationers were seasonal and in the labor or service industry. Some identified temporary agencies as a way to help find available jobs.

*“I have my daughter, my baby’s mom…Like it’s a financial situation, I guess, because we live with her mom. So, then that’s the other thing about that jobs and stuff…you’re limited to minimum wage because you got a felony and things like that…So you’re stuck just doing that much so you can only support that much. And it gets frustrating and tiresome…You work so much and you only make a little bit of money and you’re stressed out because your priorities come first and you don’t got time for you...Going through just like, and it’s like poverty too, you know what I mean? It’s just where we live right now. It’s all it is. And then, you know, you don’t go to school, get a trade or something. You’re limited to just a job.”—Black male, 24 years old*

Some participants were candid about having to resort to alternative forms of income, such as selling drugs or working “under the table”, in order to have money to provide for themselves and their families.

*“Give somebody a chance that has committed a crime…I’m more focused on selling drugs because that’s what people sell drugs for. Is to take care of themselves. You’d think it is bad…It is messed up but if you really look at the bigger picture of why the person is doing what they’re doing, it’s to feed their families. Maintain their families. Pay bills and this and that. Because that’s what I get in trouble for, selling drugs.”—Latina female, 25 years old*

*“Well, I do like little odd jobs, demolition, you know? Just because I don’t have a job really. But that slows you up, you know? It’s not all the time you can get a demolition job or you know, something to do on the side.”—Black male, 26 years old*

Six participants were employed prior to being on probation and reported that their probation status did not impact their standings with their current jobs (another theme). Two employed participants described the challenge of complying with regular probation and court visits while working. Flexibility on the part of employers was needed for time off to meet probation regulations which consist of frequently scheduled visits and time away from work. Some participants described feeling fortunate to have individuals in the community that gave them a second chance and hired them.

*“A lot of them, I filled out the application. They saw my credits. I had plenty of references and once they saw I had a B&E [breaking and entering] on my record, they said you can’t work here. So luckily, [masked] is actually a recovered addict and he saw I called him a few times and he said, ‘I’ll give you a shot.’ And it’s been five years now.”—White male, 32 years old*

#### Housing

Prominent themes that emerged from the data regarding housing included: 1) a major housing dilemma, in which housing was too expensive for individuals to afford on their own, while affordable housing puts individuals back into high-risk areas, 2) barriers to obtaining safe housing with a criminal record. Many shared stories of their rejected housing applications when landlords learned of their probation status.

Several participants stated that housing was a priority because they wanted to have their own place. A number of participants reported that they lived with parents, other family members, or friends because they could not afford to live on their own. Still others mentioned that housing was a priority because they were currently homeless or lived in places where they did not feel safe.

*“Yeah, it’s hard to be living with somebody. I’m 48 years old. I should have my own apartment.”—White female, 48 years old*

*“I’m right in the belly of the crack world, man. The apartment I live in, it’s 12 units in there, and every one of them has something to do with drugs one way or another. You got crack dealers upstairs. You got reefer dealers upstairs down the back. You got all kinds of drugs. Anything you want. You can buy a gun out of the building if you want. That’s how crazy it is. That’s why I don’t want to be there. Mentally, it’s bothering me. But the reason why I took the place is my brother gave me the money in November because it was getting cold out. I was homeless. I was in the car and stuff so he gave me the money to move in there.”—White male, 58 years old*

#### Food intake

Sixteen (73%) of the participants reported receiving monthly food stamps to assist with food intake. Key themes about food intake included: 1) rising costs of food, 2) that food stamps were not sufficient to cover their monthly food needs, and 3) self-derived mechanisms to compensate when there was not enough food.

*“A few times a month [he goes hungry]. Yeah, sometimes, you know, I’m on a bus or bike path and you miss the soup kitchen or something like that. A lot of the time food stamps just don’t last. A lot of times I go without. But I don’t ask. I don’t beg. I don’t stand on the corner holding signs, ‘Homeless.’”—White male, 58 years old*

*“Sometimes I eat a little. Give everything to my kids.”—Latino male, 29 years old*

Ten of the 16 participants (63%) receiving food stamps benefits stated that the amount of food stamps they received each month was not sufficient to meet their food needs.

*“You got $194 you get a month… So $194, you’re looking at $97 every two weeks. You’re looking at about $48.50 a week. And that’s like $6 and something a day. $7. A little less than $7 a day.”—Black male, 29 years old*

*“No because I end up using some of my pay, too, at the end of the month. Probably about $80 [of her own paycheck each month]. So it’s not a lot but it is a lot when you’re working part time.”—White female, 38 years old*

### Healthcare and current health

Participants ranked healthcare as their lowest priority. Nine individuals (41%) reported having a primary care physician they see regularly. Forty-one percent of the participants reported having state insurance through the Affordable Care Act; 27% had Medicare, Medicaid, or a combination of both; one participant had private insurance through work; one was not sure whether he had health insurance; and two reported not having health insurance. Three participants did not report their health insurance status.

A majority of probationers with state insurance reported that they were relieved to have insurance, but identified some gaps in what was covered and noted that they were required to pay high deductibles. These shortcomings represent potential barriers to accessing healthcare.

*“I know this was huge, trying to find healthcare just not even probation-wise…and then if you don’t have insurance, then you get flagged at the end of the year and have to pay. So either way, I’m going to have to pay now out-of-pocket and not go to the doctor’s…Because I can’t afford to go to the doctor’s even if I have health care insurance.”—White female, 30 years old*

*“Ever since the Obama care thing came in. He helped us but he ruined it…it helped a lot of people who didn’t have insurance, don’t get me wrong…I’m in an IOP [Intensive Outpatient Program] right now and I only have 11 sessions. Supposed to be 24 because of ever since that new Obama thing, they want to cover only a month of whatever it is. Counseling this or that. You got copays on your meds now.”*

All of the participants disclosed their current health status. Participants reported having diabetes (18%), high blood pressure (18%), high cholesterol (18%), mental health disorder (18%), asthma (9%), hepatitis C (9%), and stroke (5%). Of the probationers that reported having diabetes, high cholesterol, and high blood pressure, three reported having more than one condition and two reported having all three. Fourteen (64%) of the participants had BMIs categorized as overweight or obese. One female was pregnant during the interview so her BMI was not calculated. However, only two participants perceived themselves as overweight and were concerned with heavier weight putting them at increased risk for diabetes. Six individuals were concerned about a family history of diabetes, one individual was worried about a family history of high cholesterol and cancer, and six individuals (27%) reported having no current health issues.

## Discussion

To our knowledge, our qualitative study is the first to explore priorities necessary for subsistence among probationers. Our study highlights the competing priorities between securing basic needs and obtaining adequate healthcare among adults on probation due to material hardships, constrained resources, and their criminal justice involvement. Participants ranked substance use recovery, employment, housing, and food intake as the top four priorities, well before healthcare. While only a small number of participants reported being overweight or having a chronic disease, many presented profiles that put them at risk for future chronic disease outcomes. Furthermore, several participants indicated that they were not regularly accessing healthcare, which may result in an underestimation of actual diagnoses. According to the 2011–12 National Inmate Survey, prisoners and jail inmates had a significantly higher prevalence of chronic disease compared to the general population [7]. There are no data available on chronic disease rates for probationers.

Food intake, although ranked fourth, was the issue most frequently selected as a priority among participants. Food insecurity has not been widely studied in probationers. In other similar populations, such as people who were homeless or using substances, food insecurity has been associated with risky sexual practices and obesity [12, 27–29]. Two-thirds of participants in the present study were overweight or obese, which is similar to the general population [30]. In addition, for individuals who are food insecure, dietary quality is likely to be poor, further exacerbating the risk for developing chronic disease.

Participants provided examples of how the top ranked priorities were interrelated and the detrimental impacts on their health, including mental health. During discussions about employment barriers, for example, participants also discussed difficulties with accessing adequate amounts of food and safe housing. Participants described how the low wage jobs available for probationers would not be sufficient to meet basic needs or bring them out of poverty. Substance use recovery was identified as the highest ranking priority for the fewest number of participants; several individuals described their need for sobriety in order to escape the problems associated with addiction. Yet, several participants reported living in neighborhoods with high crime and drug use and some indicated their need to sell illicit drugs for income to support their families given the limited options for employment and government support. These structural barriers shape where probationers live and often determine their access to health-related resources. In addition, testimonies of perceived stigma and discrimination in housing and employment were often shared. The marginalization of this population and their perceptions of stigma and discrimination magnify the chronic “weathering” [31] individuals under correctional supervision endure, which further compromises health. In addition, our study highlights that meritocracy, the belief of achieving the American dream and economic success based on an individual’s work ethic and resilience [32], is elusive for individuals in the criminal justice system. Being under correctional supervision is the punishment for the felony they committed, but participants often experienced punitive attitudes and mistrust in their encounters with others, which posed challenges with their re-integration into society.

Our study findings should be considered in light of several limitations. In-depth interviews were conducted at the probation office, which may have contributed to reserved responses and underreporting of illicit behaviors, especially with regard to current substance use. We believe the lack of disclosure about current substance use did not spill over into other reporting as participants appeared to be comfortable discussing the other topics fully, including prior histories of substance use. Although we acknowledge that our sample size was a relatively small sample size, it appeared to be of sufficient size to address our research questions [33, 34]. Data were collected at one probation office in Rhode Island and viewpoints may not represent all probationers in the state. The interpretations of the findings were not triangulated with the probation population, however, probation officers confirmed our findings, adding credibility.

## Conclusions

To our knowledge, this is the first study to represent probationer perspectives and experiences with regard to life and health priorities, and can serve as an initial point of entry for future research in this area. The narratives from our study suggest that many probationers with best intentions to start a new life path were often thrust back into an environment with poverty, drug use, drug sales, and crime in close proximity to or within family and peer networks that span across generations. These cyclical challenges with limited economic potential and support systems constantly place probationers at high risk for recidivism or extended lengths of their community correctional supervision. These findings help to inform the context of the marginalized circumstances individuals on probation encounter and emphasize the need for structural changes, and better targeting of interventions that correspond with probationer priorities, to begin to mitigate health disparities in this population. Future research, programming, and policies should focus on the priorities identified by this population, including the availability of substance use recovery treatment programs, gainful employment opportunities, safe housing, and adequate food. These basic needs are the foundations for achieving wellbeing and without confronting the barriers to meeting these basic needs, healthcare interventions may be futile.

## Abbreviations

BMI: Body mass index
IOP: Intensive outpatient program
